# Distinct OGT-Binding Sites Promote HCF-1 Cleavage

**DOI:** 10.1371/journal.pone.0136636

**Published:** 2015-08-25

**Authors:** Tanja Bhuiyan, Patrice Waridel, Vaibhav Kapuria, Vincent Zoete, Winship Herr

**Affiliations:** 1 Center for Integrative Genomics, University of Lausanne, Génopode, Lausanne, Switzerland; 2 Protein Analysis Facility, Center for Integrative Genomics, Faculty of Biology and Medicine, University of Lausanne, Génopode, Lausanne, Switzerland; 3 Molecular Modeling Group, Swiss Institute of Bioinformatics, Génopode, Lausanne, Switzerland; Stanford University, UNITED STATES

## Abstract

Human HCF-1 (also referred to as HCFC-1) is a transcriptional co-regulator that undergoes a complex maturation process involving extensive *O*-GlcNAcylation and site-specific proteolysis. HCF-1 proteolysis results in two active, noncovalently associated HCF-1_N_ and HCF-1_C_ subunits that regulate distinct phases of the cell-division cycle. HCF-1 *O*-GlcNAcylation and site-specific proteolysis are both catalyzed by *O*-GlcNAc transferase (OGT), which thus displays an unusual dual enzymatic activity. OGT cleaves HCF-1 at six highly conserved 26 amino acid repeat sequences called HCF-1_PRO_ repeats. Here we characterize the substrate requirements for OGT cleavage of HCF-1. We show that the HCF-1_PRO_-repeat cleavage signal possesses particular OGT-binding properties. The glutamate residue at the cleavage site that is intimately involved in the cleavage reaction specifically inhibits association with OGT and its bound cofactor UDP-GlcNAc. Further, we identify a novel OGT-binding sequence nearby the first HCF-1_PRO_-repeat cleavage signal that enhances cleavage. These results demonstrate that distinct OGT-binding sites in HCF-1 promote proteolysis, and provide novel insights into the mechanism of this unusual protease activity.

## Introduction

Post-translational modifications can alter protein functions and thus greatly enhance the diversity of gene function. There are two principal types of post-translational protein modifications: proteolysis, which is essentially irreversible, and attachment of a chemical group—such as glycosylation studied here—which is often reversible.

One of the most abundant forms of glycosylation in eukaryotic organisms is the addition of N-acetylglucosamine (GlcNAc) to serines or threonines of nuclear and cytoplasmic proteins, giving rise to *O*-linked β-N-acetylglucosamine (*O*-GlcNAc) proteins [1]. The enzyme catalyzing this modification is the highly conserved glycosyltransferase *O*-linked β-N-acetylglucosamine transferase (OGT), which uses the sugar donor UDP-GlcNAc as cofactor. UDP-GlcNAc is the end product of the hexosamine biosynthetic pathway, a nutrient-sensing pathway, and *O*-GlcNAcylation has thus been proposed to reflect cellular nutrient levels [2, 3]. *O*-GlcNAcylation can affect protein phosphorylation [4], enzymatic activity [5], protein stability [6], specific protein-protein interactions [7] or protein aggregation [8]. We have recently shown that OGT also possesses an unexpected proteolytic activity towards the human host-cell factor 1 (HCF-1) [9].

HCF-1 is a transcriptional co-regulator that is not only cleaved [9], but also *O*-GlcNAcylated [10–12] by OGT. After synthesis as a 2035 amino acid precursor protein, HCF-1 undergoes a maturation process involving *O*-GlcNAcylation of the N-terminal half of the protein [9, 12] and site-specific proteolysis, resulting in a set of N- (HCF-1_N_) and C- (HCF-1_C_) terminal subunits [9, 10, 13, 14]. After processing, the HCF-1_N_ and HCF-1_C_ subunits remain tightly, but noncovalently associated [13–15] and regulate distinct phases of the cell-division cycle [16]. HCF-1_N_ promotes G1-to-S-phase transition [16] via association with E2F transcription factors [17, 18] and Thap11 [19], and HCF-1_C_ promotes proper M-phase progression [16]. OGT-mediated HCF-1 proteolytic processing ensures proper cell-cycle progression through activation of HCF-1_C_-subunit M-phase functions [9].

HCF-1 proteolysis occurs at six centrally located and highly conserved repeated sequences called HCF-1_PRO_ repeats. Each HCF-1_PRO_ repeat contains 26 amino acids with a conserved 20 amino acid core sequence shared among vertebrate species, but not in invertebrate species [9, 10, 14]. The HCF-1_PRO_ repeat forms an unusually large protease recognition sequence [9, 13], displaying a bipartite sequence architecture: a “cleavage region” containing a critical glutamate residue at the site of cleavage and a “threonine region” containing a number of highly conserved threonine residues.

The structure of human OGT is also bipartite with an N-terminal superhelical 13.5 tetratricopeptide-repeat (TPR) domain [20] followed by a globular catalytic domain, which contains the UDP-GlcNAc binding pocket [21]. A crystal structure of human OGT containing 4.5 TPRs in complex with an HCF-1_PRO_ repeat and a non-hydrolysable form of UDP-GlcNAc [22] has revealed that the bipartite structures of OGT and of the HCF-1_PRO_ repeat interact with each other in a complementary manner. The HCF-1_PRO_-repeat threonine region binds stably to the OGT TPR domain through a network of hydrogen bonds connecting the HCF-1_PRO_-repeat threonine side-chains and backbones with OGT, whereas the HCF-1_PRO_-repeat cleavage region binds to the OGT catalytic domain, analogous to an *O*-GlcNAcylation substrate [22]. How the OGT catalytic domain, UDP-GlcNAc and the HCF-1_PRO_ repeat promote proteolysis is not understood. Interestingly, the E10 cleavage-site residue forms an N-terminal pyroglutamate after cleavage *in vitro* that probably represents a product of the cleavage reaction [22]. Lazarus et al. [22] proposed that the cofactor UDP-GlcNAc plays a pivotal role for cleavage, because (i) it is located in close proximity to the E10 side-chain at the cleavage site, and (ii) it is strictly required for HCF-1_PRO_-repeat proteolysis.

In the present study, we analyze HCF-1–OGT interactions that promote proteolysis. We probe how the HCF-1_PRO_ repeat interacts with OGT and UDP-GlcNAc and thus extend our understanding of the cleavage mechanism. Furthermore, we identify a novel OGT-binding sequence nearby the first HCF-1_PRO_ repeat that enhances HCF-1_PRO_-repeat cleavage. Thus, multiple distinct OGT-binding sites in HCF-1 promote HCF-1 cleavage.

## Results

### The glutamate residue at the HCF-1_PRO_-repeat cleavage site displays an unfavorable binding behavior to OGT

**Fig 1:**
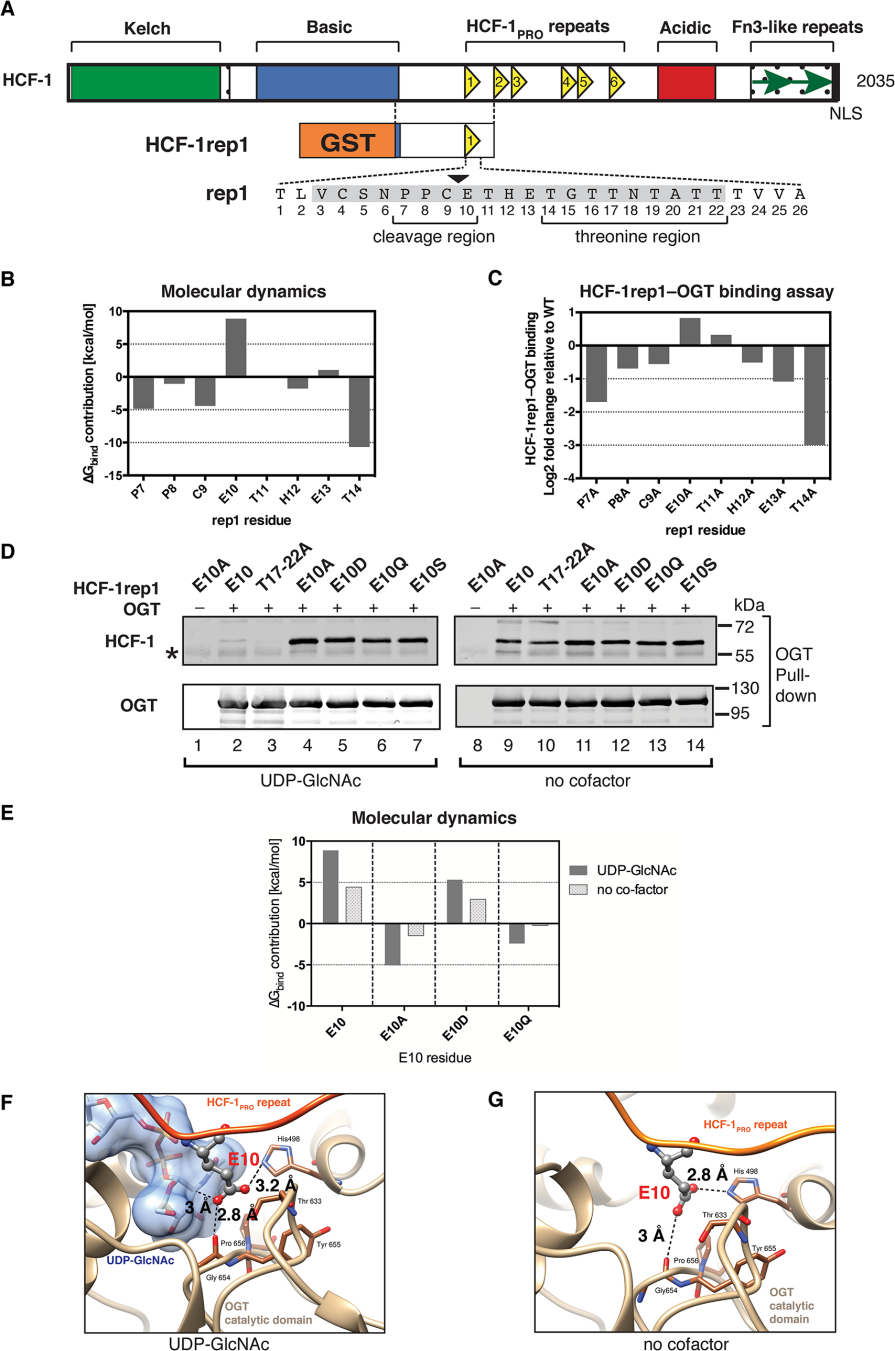
The glutamate residue at position 10 in the HCF-1_PRO_ repeat inhibits OGT binding. (A) (Top) Schematic representation of the human HCF-1 protein showing the six HCF-1_PRO_ repeats as yellow triangles. (Bottom) Schematic of the recombinant HCF-1rep1 precursor (HCF-1 residues 867–1071), containing the HCF-1_PRO_ repeat 1 (rep1) and surrounding sequences fused to glutathione-S-transferase (GST). The 26 residues of rep1 are shown and the site of proteolysis is marked at the C/E sequence by a black arrowhead. The 20 amino acid core sequence [14] is shaded gray, and the cleavage and threonine regions are indicated. (B) E10 exhibits inhibitory contributions to OGT binding. Computational estimation of the contributions to the binding free-energy, Δ*G*_*bind*_, of single amino acid residues in the HCF-1_PRO_ repeat (P7-T14) for the association with OGT. Negative and positive values correspond to favorable and unfavorable OGT-association contributions, respectively, in the *in silico* structural complex (based on the 4N3B structure [22]). (C) An E10 to alanine substitution enhances HCF-1_PRO_-repeat–OGT binding. *In vitro* HCF-1rep1–OGT binding assay in the presence of UDP-GlcNAc with HCF-1rep1 constructs containing individual alanine substitutions in the HCF-1_PRO_ repeat (P7A-T14A). HCF-1rep1 binding to OGT was quantified from an immunoblot (S1B Fig) as ratio of OGT-bound HCF-1rep1 to total HCF-1rep1 in the assay. Obtained values are presented as log2 fold change relative to wild-type HCF-1rep1–OGT binding. (D) HCF-1_PRO_-repeat–OGT binding is influenced by the E10 side-chain properties and exhibits sensitivity to UDP-GlcNAc. *In vitro* HCF-1rep1–OGT binding assay. Constructs with wild-type (WT), E10 missense mutations (E10A, E10D, E10Q, and E10S), and T17-T22A mutations were incubated in the presence (left panel) or absence (right panel) of UDP-GlcNAc. Detection of OGT and HCF-1rep1 was performed using the indicated antibodies. *, IgG heavy chain. (E) Computational estimation of the contributions to the binding free-energy, Δ*G*_*bind*_, of single amino acid residues at the E10 cleavage site for the association with OGT. Shown are residue contributions of WT (E10), alanine (E10A), aspartate (E10D) or glutamine (E10Q) side-chains in the presence or absence of UDP-GlcNAc in the *in silico* structural complex (based on the 4N3B structure). (F) Close-up view (4N3C crystal structure [22]) of the OGT active site with the HCF-1_PRO_ repeat and UDP-GlcNAc. The deprotonated E10 oxygen atom exhibits an unfavorable interaction with OGT. (G) Snapshot from a molecular dynamics simulation (based on the 4N3B structure) of the OGT active site in complex with the HCF-1_PRO_ repeat without UDP-GlcNAc. The displayed frame is representative of the average distances sampled along the simulations. In (F) and (G), the E10 side-chain is shown in ball and stick representation (carbons: gray, nitrogen: blue, oxygens: red), and dashed lines indicate distances between atoms.

Previous *in vivo* and *in vitro* cleavage studies of the HCF-1_PRO_ repeat embedded in a heterologous context (i.e., the Oct-1 transcription factor) have shown that the activities of the cleavage and threonine regions are sensitive to alanine substitutions [9, 13]. To probe the role of HCF-1_PRO_-repeat residues for cleavage in their natural sequence environment, we used a recombinant cleavage substrate called HCF-1rep1 [9]. Fig 1A illustrates the structure of human HCF-1 with the six HCF-1_PRO_ repeats shown in yellow. The HCF-1rep1 substrate, shown below the full-length HCF-1 structure, contains the first HCF-1_PRO_ repeat embedded into its HCF-1 context. We performed *in vitro* cleavage assays with bacterially purified human OGT and HCF-1rep1 substrates with residues surrounding the cleavage site (P7-T14) mutated to alanine. The results (S1A Fig) paralleled those of the previous Oct-1/HCF-1 hybrid studies [9, 13], although the HCF-1_PRO_-repeat residues T11, H12, and E13 exhibited little importance in this assay, leading us to refine the HCF-1_PRO_-repeat cleavage region to the sequence encompassing residues P7 to E10, as shown in Fig 1A.

*In vivo* co-immunoprecipitation assays have shown that the replacement of the E10 residue by alanine (E10A) enhances HCF-1–OGT association [9]. To characterize this and other interactions between the HCF-1_PRO_-repeat cleavage region and the OGT catalytic domain, we first estimated the individual side-chain contributions to OGT binding for residues P7 to T14, using the molecular mechanics–generalized born surface area (MM-GBSA) approach [23, 24]. For this purpose, we used the HCF-1_PRO_-repeat–OGT crystal structure (PDB code 4N3B [22]) and replaced the E10Q glutamine residue in the HCF-1_PRO_ repeat of that structure by glutamate and UDP-5SGlcNAc (a non-hydrolysable cofactor used for structure determination [22, 25]) by UDP-GlcNAc. We note that the configurations of the glutamate (E10) in the replacement *in silico* structure and the glutamine (E10Q) side-chains in the complex are nearly identical [22]. These computational calculations predicted that the E10 residue is indeed unusual within the cleavage region: it is the only residue between P7 and T14 to display highly unfavorable interactions with OGT complexed with UDP-GlcNAc (Fig 1B). In contrast, T14 of the threonine region and P7 and C9 of the cleavage region display favorable OGT-binding contributions and, consistent with the aforementioned cleavage assay (S1A Fig), the intervening residues (T11–E13) exhibit minor or no contributions to OGT binding.

To support the results obtained by the molecular dynamics approach, we tested the individual P7 to T14 HCF-1_PRO_-repeat alanine mutants in an *in vitro* HCF-1rep1–OGT binding assay in the presence of UDP-GlcNAc, as described [22]. Mutant HCF-1rep1 binding to OGT was quantified and normalized to wild-type HCF-1rep1 binding (Fig 1C) from an immunoblot analysis shown in S1B Fig. Consistent with the molecular dynamics results, only the E10A mutation caused a considerable increase in HCF-1rep1 binding with respect to wild-type (Fig 1C). Because other point mutations, such as P8A and C9A inhibit proteolysis (S1A Fig), but do not promote OGT binding, the increase in OGT affinity of the E10A mutant is not simply explained by an inhibition of proteolysis of the binding substrate. Thus, physical interaction assays, as well as computational analyses indicate that E10 possesses unfavorable interactions in the OGT–UDP-GlcNAc–HCF-1_PRO_-repeat complex.

### E10 side-chain incompatibility in the OGT–UDP-GlcNAc–HCF-1_PRO_-repeat complex

The observation that E10 is the only residue that we tested in the HCF-1_PRO_ repeat that interferes with binding to OGT and is positioned near the sugar moiety of UDP-GlcNAc in the crystal structure [22] led us to ask whether this E10 property is dependent on the presence of UDP-GlcNAc. For this purpose, we performed *in vitro* binding assays with a set of HCF-1rep1 mutants containing E10 substitutions (E10A, E10Q, E10D, and E10S) that have been described to inhibit HCF-1_PRO_-repeat cleavage [22]. The E10S mutant not only inhibits cleavage like E10A, E10Q, and E10D, but also provides an active substrate for HCF-1_PRO_-repeat *O*-GlcNAcylation [22]. Fig 1D shows HCF-1rep1–OGT binding in the presence (lanes 1–7) or absence (lanes 8–14) of UDP-GlcNAc. As previously described [22], in the presence of UDP-GlcNAc, the wild-type E10 substrate displayed reduced OGT binding (Fig 1D, lane 2), whereas the E10A substrate displayed enhanced OGT binding (lane 4). Similarly to the E10A mutant, the E10D and E10Q mutants (lanes 5 and 6) enhanced binding to OGT, in spite of the structural similarities between the glutamate, glutamine, and aspartate side-chains. The E10S *O*-GlcNAcylation substrate enhanced OGT binding (lane 7) like the E10A, E10Q, and E10D mutants. Thus, replacement of E10 by either similar (E10Q and E10D) or unlike (E10A and E10S) residues increased OGT binding. The threonine region T17–22A mutant contains four grouped threonine to alanine substitutions and was shown to inhibit binding to the OGT TPR domain [22]. As previously described [22], in the presence of UDP-GlcNAc, the T17–22A mutant displayed reduced OGT binding (lane 3).

When UDP-GlcNAc was omitted from the assay, however, binding of the E10 missense mutants was not noticeably altered (lanes 11–14), whereas binding of the substrates containing E10 in their HCF-1_PRO_ repeats (WT and T17–22A) increased considerably (Fig 1D, compare lanes 9 and 10 to lanes 2 and 3). These results suggest that UDP-GlcNAc exhibits a negative effect on binding of the HCF-1_PRO_-repeat E10 residue in the OGT active site and that this effect is highly dependent on the glutamate structure. The inhibitory effect also depends on the sugar moiety of UDP-GlcNAc, as with UDP alone, the E10A mutant did not enhance OGT association (S1C Fig).

To determine the role of the E10 side-chain for interactions with the OGT–UDP-GlcNAc complex, we estimated the OGT-binding contributions of E10, E10A, E10D, and E10Q residues in the presence or absence of UDP-GlcNAc using molecular modeling, as described above. In this analysis (Fig 1E), in the presence of UDP-GlcNAc, the wild-type E10 residue displayed the most unfavorable OGT interaction among the tested residues, as indicated by the positive value for the contribution to the binding free-energy (∆G_*bind*_). E10D displayed a more favorable OGT interaction, albeit still inhibitory, presumably because of the similarly negatively charged aspartate side-chain. The neutral side-chains of E10A and E10Q both displayed favorable interactions with OGT. When UDP-GlcNAc was omitted from the OGT active site in the molecular dynamics simulations, the contributions to the binding free-energy of the unfavorable E10 and E10D contacts decreased, suggesting that the removal of UDP-GlcNAc can release strains in the complex, consistent with the *in vitro* binding assays (Figs 1C, 1D, and S1C). We conclude that the inhibitory effect of the E10 side-chain is most prominent in the presence of UDP-GlcNAc. Thus, not only HCF-1_PRO_-repeat proteolysis but also HCF-1_PRO_-repeat–OGT association is influenced by the E10 side-chain structure and UDP-GlcNAc—perhaps this specific type of HCF-1_PRO_ repeat and OGT association promotes cleavage.

To understand the E10 interaction better, we examined the location of the HCF-1_PRO_-repeat E10 side-chain and UDP-GlcNAc in the OGT active site of the crystal structure (PDB code 4N3C [22]). Consistent with the above-described results, we noticed that the E10 side-chain is located in close proximity (2.8 Å) of the carbonyl oxygen of glycine 654 in the OGT catalytic domain, potentially causing an unfavorable electrostatic repulsion (Fig 1F). The E10 carboxylate functional group is maintained in this unfavorable position by the glucose moiety of UDP-GlcNAc. When we performed a molecular dynamics simulation without UDP-GlcNAc (representative snapshot along the trajectory shown in Fig 1G), the E10 residue can change its position to prevent the unfavorable interaction with glycine 654 and appears to form a favorable interaction with a nitrogen atom in the imidazole ring of histidine 498 in the OGT catalytic domain.

Together, these results suggest that UDP-GlcNAc inhibits HCF-1_PRO_-repeat–OGT association by imposing that the E10 side-chain be in an unfavorable position within the complex. This incompatibility may cause strains in the OGT–UDP-GlcNAc–HCF-1_PRO_-repeat complex, which, we hypothesize, initiates or facilitates HCF-1_PRO_-repeat proteolysis. We thus propose that the mechanism of OGT-mediated HCF-1 proteolysis involves strains originating from the cleavage substrate.

### Sequences lying outside of the HCF-1_PRO_ repeat promote proteolysis

**Fig 2:**
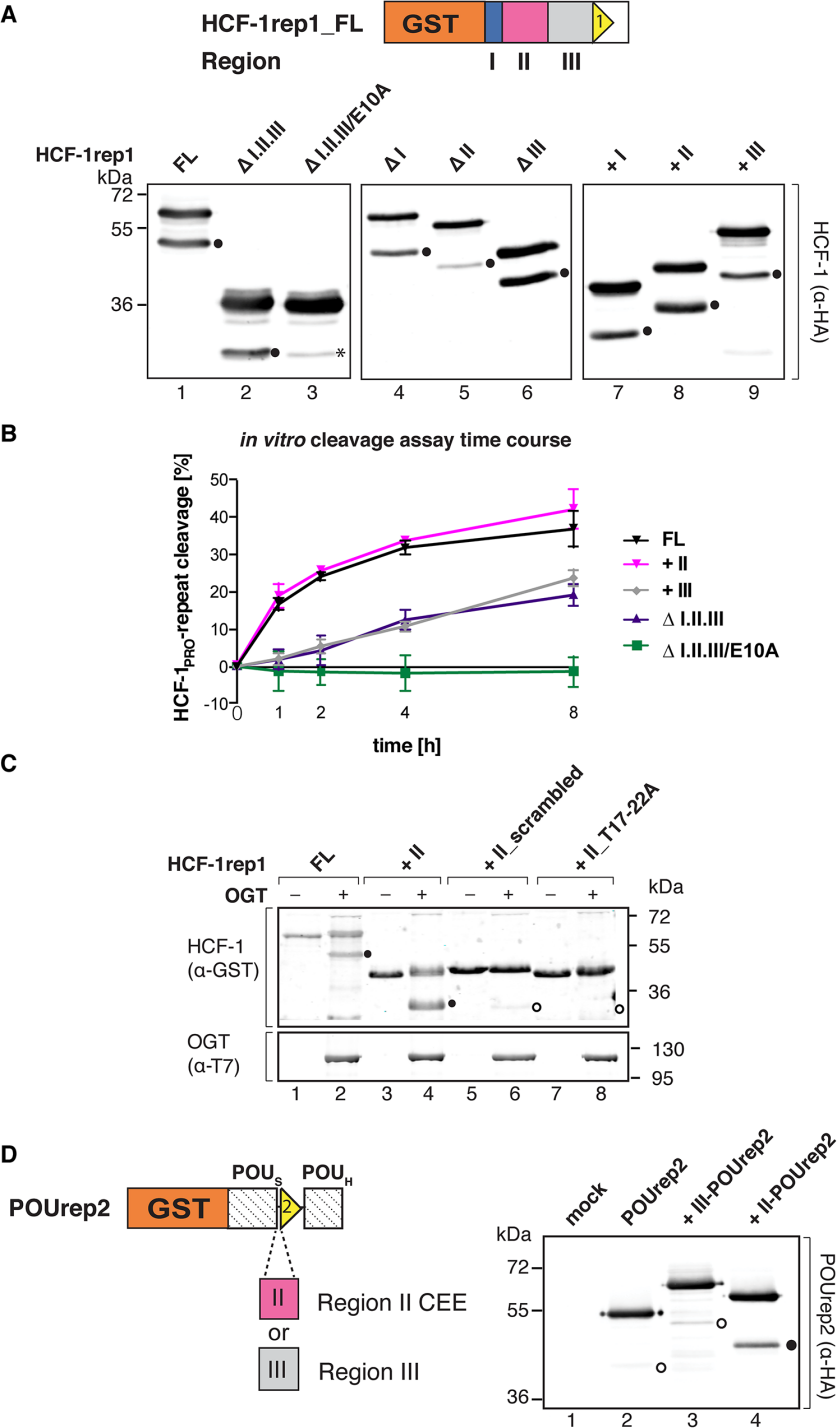
HCF-1_PRO_-repeat cleavage enhancement by a sequence nearby the HCF-1_PRO_ repeat 1. (A) HCF-1_PRO_-repeat cleavage is context dependent *in vivo*. (Top) Schematic of the HCF-1rep1 precursor subdivided into Region I (25 residues, blue), Region II (58 residues, pink), and Region III (60 residues, gray). (Bottom) HEK 293 cells were transfected with expression vectors encoding HCF-1rep1 FL or deletion constructs, either containing or lacking Regions I, II or III. Proteins were immunoprecipitated by an N-terminal HA-tag and assayed for cleavage by visualization by α-HA-tag immunoblot. *, C-terminal precursor truncations. (B) Region II enhances HCF-1_PRO_-repeat cleavage *in vitro*. Cleavage efficiency during an *in vitro* cleavage assay time course of selected HCF-1rep1 constructs. HCF-1rep1 constructs were incubated with OGT for 0 to 8 h and precursor and resulting N-terminal cleavage products were analyzed for cleavage by α-GST-immunoblot. Uncleaved and cleaved products were quantified and cleavage efficiencies determined as cleaved products over total. Shown are the means and standard deviations of three independent experiments. (C) Region II cleavage-enhancement activity is sequence specific. *In vitro* cleavage assay of HCF-1rep1 FL and Region II constructs containing a scrambled Region II sequence (+II_scrambled) or an inactive HCF-1_PRO_ repeat (+II_T17–22A). Resulting precursor and N-terminal cleavage products were analyzed for cleavage with the indicated antibodies. (D) Region II activates the inactive POUrep2 construct for cleavage. (Left) Schematic of the GST-fusion construct POUrep2 containing HCF-1_PRO_ repeat 2 (rep2), embedded in between the POU-specific (POU_S_) and POU-homeo domains (POU_H_) of Oct-1. Region II or Region III were inserted N-terminal of rep2, respectively. (Right) *In vivo* cleavage activities in HEK 293 cells, transiently transfected with transfection medium (mock) or POUrep2 encoding plasmids. Precursors and cleaved fragments were purified via immunoprecipitation of an N-terminal HA-tag and cleavage assayed using the indicated antibody. In (A), (C) and (D), prominent (●) and faint (⭕) cleavage products are indicated.

Unexpectedly, in previous experiments, a single HCF-1_PRO_ repeat was not sufficient for OGT-mediated proteolysis (Capotosti and Herr, unpublished results). As the HCF-1rep1 substrate (Fig 1A) was cleaved, we asked what sequences outside of the HCF-1_PRO_ repeat in this construct might be promoting cleavage. For this purpose, we divided the residues upstream of HCF-1_PRO_ repeat 1 into three regions (Fig 2A): Region I, comprising identified *O*-GlcNAcylation sites [9], and Region II and Region III, which contain nearly the same number of residues, 58 and 60, respectively. We note that the sequences of these three regions are conserved in vertebrate species (S2A Fig; [26]), where HCF-1 cleavage is meditated by OGT, but not in invertebrate species, where HCF cleavage is mediated by Taspase1 [27]. We generated deletion constructs (schematics in S2B Fig) containing only one of the three Regions I-III (referred to as +I, +II, and +III), lacking only one of the three regions (referred to as ∆I, ∆II, and ∆III) or lacking all three regions together (called ∆I.II.III). The E10A mutation in ∆I.II.III/E10A served as a negative control for cleavage.

In a 48-hour *in vivo* transient transfection HCF-1rep1 cleavage assay (Fig 2A), the absence of all three regions together lowered HCF-1_PRO_-repeat cleavage efficiency when the ratios between cleaved product and total of uncleaved and cleaved products of each construct were compared (Fig 2A, lanes 1–3). Among the three regions, the lack of Region II had the strongest negative effect on cleavage (lanes 4–6). The addition of Regions I, II, and III individually enhanced HCF-1_PRO_-repeat cleavage (lanes 7–9) with respect to the ∆I.II.III construct (lane 2), with Region II, offering the greatest enhancement and Region III promoting minimal enhancement. This *in vivo* HCF-1 cleavage assay displayed variability among independent experiments, particularly in reference to the ∆I.II.III and ∆III constructs (S2C Fig), most likely owing to the difficulty in controlling *in vivo* synthesis rates and stability of the different constructs via the transient transfection assay. Nevertheless, these results led us to conclude that, although all three regions display cleavage-enhancement activity, Region II has the most pronounced activity on HCF-1_PRO_-repeat proteolysis.

### An HCF-1 cleavage enhancer element (CEE) promotes efficient HCF-1_PRO_-repeat proteolysis

To solidify the aforementioned conclusion concerning the *in vivo* cleavage studies, we performed *in vitro* cleavage assays under controlled conditions with bacterially synthesized proteins and determined the rates of cleavage of a subset of HCF-1rep1 constructs in an eight-hour time course. The rate of cleavage for the ∆I.II.III construct was substantially lower than for the full-length (FL) construct (Fig 2B). Relative to these activities, the FL and +II HCF-1rep1 constructs displayed similarly enhanced activities, whereas the +III and ∆I.II.III constructs displayed similarly reduced activities. The difference between the two groups was particularly evident at the earlier time points, i.e., after 1 and 2 hours. Together with the *in vivo* studies above, these results indicate a robust role for Region II in HCF-1_PRO_-repeat cleavage enhancement.

To probe the sequence dependence of the activity of Region II on cleavage, we replaced the wild-type sequence in construct +II by a scrambled Region II sequence (+II_scrambled). As shown in Fig 2C, in a 4-hour *in vitro* cleavage assay, +II_scrambled (lanes 5 and 6) showed little cleavage activity when compared to the wild-type +II construct (lanes 3 and 4), indeed no more activity than a Region II construct with an inactive HCF-1_PRO_ repeat (+II_T17–22A) (lanes 7 and 8). This result suggests that the specific amino acid sequence of Region II plays a role in the enhancement of HCF-1_PRO_-repeat cleavage.

In previous studies, HCF-1_PRO_-repeat cleavage was assayed when the repeat was embedded within the C-terminal region of the Oct-1 POU transcription factor [9, 13]. When, however, embedded within the flexible linker [28] connecting the two structured domains of the Oct-1 POU DNA-binding domain (construct POUrep2, Fig 2D), the HCF-1_PRO_ repeat was inactive (Capotosti and Herr, unpublished results). To test whether Region II can reactivate the POUrep2 substrate, we inserted either Region II (construct +II-POUrep2) or Region III (+III-POUrep2) upstream of the inactive HCF-1_PRO_ repeat. As shown in a 48-hour *in vivo* cleavage assay (Fig 2D), the POUrep2 construct (lane 2) displayed little, if any, cleavage activity. Region III weakly enhanced HCF-1_PRO_-repeat cleavage (lane 3), whereas Region II enhanced cleavage more effectively (lane 4). Thus, Region II is a sequence-specific enhancer of HCF-1_PRO_-repeat cleavage. We therefore refer to this sequence as a Region II HCF-1_PRO_-repeat cleavage enhancer element or Region II CEE.

### The Region II CEE is a novel OGT-binding sequence

**Fig 3:**
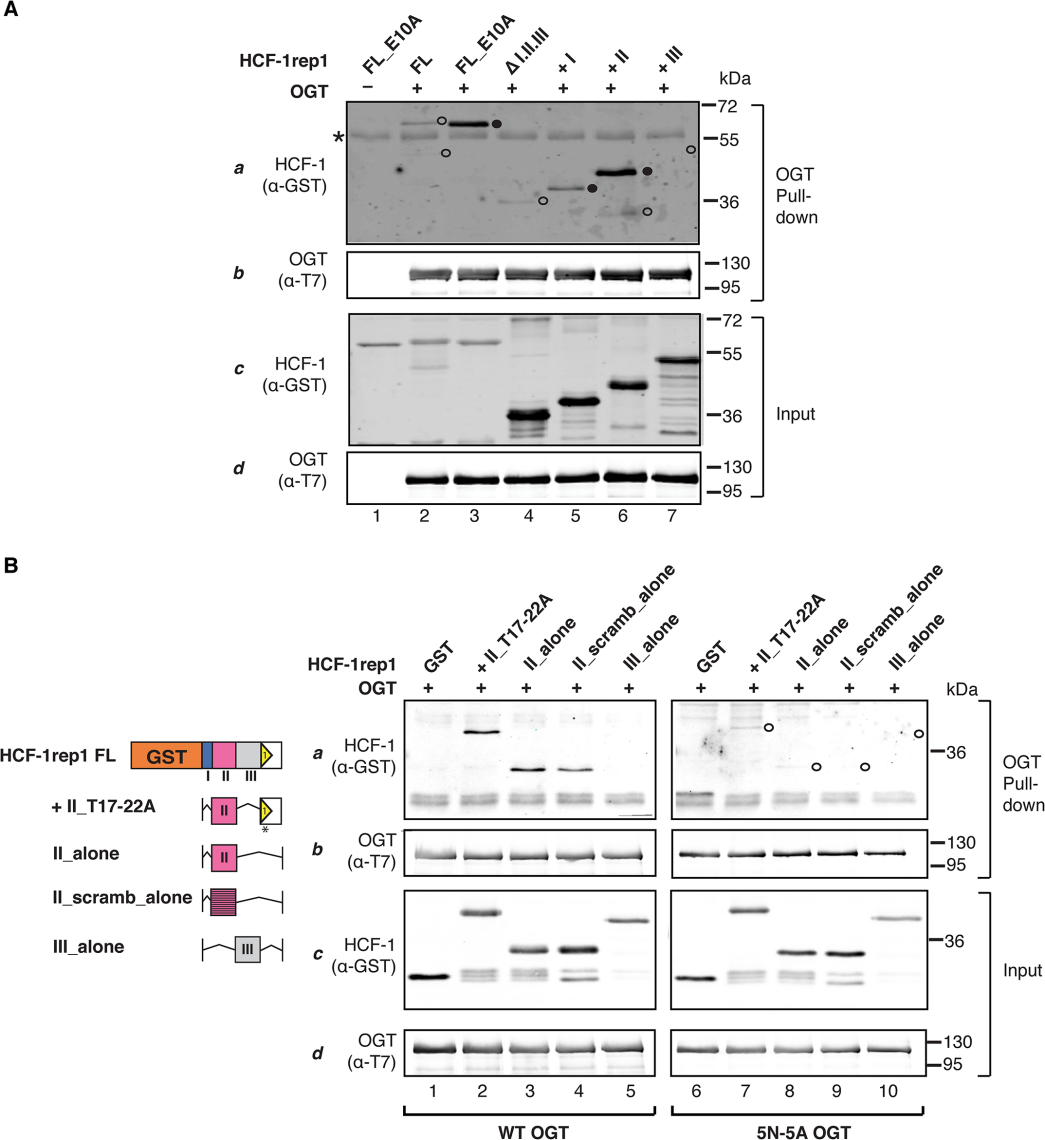
The Region II CEE represents an OGT-binding sequence. (A) Region II enhances OGT–HCF-1rep1 binding. Full-length (FL) and deletion HCF-1rep1 constructs were tested for OGT binding in the presence of UDP-GlcNAc using an *in vitro* OGT-directed pull-down assay. Detection of OGT and HCF-1rep1 was performed, using the indicated antibodies. Shown are 100% of OGT pull-down (panels a and b) and 11% of the input (panels c and d). *, IgG heavy chain. (B) HCF-1_PRO_-repeat-independent OGT–Region II binding. (Left) Schematics of the HCF-1 constructs used in this experiment. (Right) HCF-1rep1 containing Region II and an OGT-binding defective HCF-1_PRO_ repeat (+II_T17–22A), or GST-fusion constructs containing Region II (wild-type or scrambled) alone or Region III alone (II_alone, II_scramb_alone, III_alone) were tested for binding with wild-type (WT) (left panel) or 5N-5A mutant (right panel) OGT. HCF-1 binding was detected as in (A). In (A) and (B), weak (⭕) and effective (●) OGT binding is indicated.

As the Region II CEE influences HCF-1_PRO_-repeat cleavage in a sequence-dependent manner, we asked whether it represents a sequence-specific binding site for OGT. Fig 3A (panel a) shows HCF-1rep1 binding in an OGT-directed pull-down assay with a set of mutant HCF-1rep1 substrates. Because of the inhibitory role of E10 for OGT binding, full-length wild-type HCF-1rep1 (Fig 3A, FL, lane 2) bound more weakly to OGT than the E10A mutant (E10A, lane 3). The ∆I.II.III construct displayed weak OGT association (lane 4), correlating with its low cleavage efficiency, whereas, relative to the +I and +III constructs, the +II construct bound strongly to OGT (compare lane 6 to lanes 5 and 7), correlating with the cleavage activities of these constructs. These results suggest that the Region II CEE contains an OGT-binding site.

As the HCF-1_PRO_ repeat itself represents an OGT-binding site, we investigated whether the Region II CEE can bind OGT independently of the HCF-1_PRO_ repeat and whether the binding mode is similar. To test this in an *in vitro* HCF-1rep1–OGT binding assay, we employed two strategies. First, we assayed wild-type OGT binding (Fig 3B, lanes 1–5) to a set of HCF-1 constructs: a +II HCF-1rep1 construct defective for HCF-1_PRO_-repeat–OGT interactions (i.e., +II_T17–22A), or constructs with Region II (wild-type or scrambled) or Region III alone (i.e., II_alone, II_scramb_alone, or III_alone). The constructs containing the Region II wild-type sequences clearly associated with WT OGT (Fig 3B, lanes 2 and 3), showing that Region II can associate with OGT independently of the HCF-1_PRO_ repeat. Curiously, although the scrambled Region II sequence did not enhance HCF-1_PRO_-repeat proteolysis (Fig 2C), it did bind to OGT (Fig 3B, lane 4), suggesting that OGT–Region II binding possesses relaxed sequence dependency. Nevertheless, OGT–Region II binding is clearly not entirely sequence independent, as the Region III sequence did not bind efficiently to OGT (lane 5).

In the second strategy, we probed whether the OGT TPR domain is involved in OGT–Region II binding. For this purpose, we used the HCF-1 cleavage and *O*-GlcNAcylation compromised 5N-5A OGT mutant, which disrupts HCF-1_PRO_-repeat–OGT association [22]. The results show (Fig 3B, lanes 6–10) that the OGT TPR domain is important for OGT binding of the Region II sequences, suggesting a role of the OGT TPR domain in Region II recognition and binding.

We conclude that OGT binds autonomously to the Region II CEE, representing an independent OGT-binding site. Furthermore, OGT–Region II interactions are mediated by the OGT TPR domain, showing that the TPRs not only recognize the threonine region, but also other HCF-1 sequences lying outside of the HCF-1_PRO_ repeat.

### The Region II CEE contains a cluster of *O*-GlcNAcylation sites

HCF-1 is a highly *O*-GlcNAcylated protein and *O*-GlcNAcylation sites, predominantly in the HCF-1_N_ subunit, have been identified in several studies [9, 12, 29, 30]. To identify potential *O*-GlcNAcylation sites in the Region II CEE, we performed liquid chromatography followed by tandem mass spectrometry (LC-MS/MS). *O*-GlcNAcylation sites in an HCF-1 precursor construct (residues 686 to 1166) spanning the HCF-1 residues contained in the HCF-1rep1 construct have been described [9], but the HCF-1 sequences covering Region II were not included, because the peptides generated by combined trypsin and Glu-C digestion of this region were not appropriate for LC-MS/MS analysis [9]. Thus, *O*-GlcNAcylation sites were identified in Region I, but could not be in Region II and a part of Region III. To overcome the lack of peptide coverage in the Region II and III sequences for LC-MS/MS analysis, we engineered two trypsin cleavage sites by site-directed mutagenesis at two different positions within the HCF-1 sequence (A933K and M951K). The mutations had no effect on HCF-1rep1 cleavage and overall *O*-GlcNAcylation levels, as determined by immunoblot (S3A Fig).

**Fig 4:**
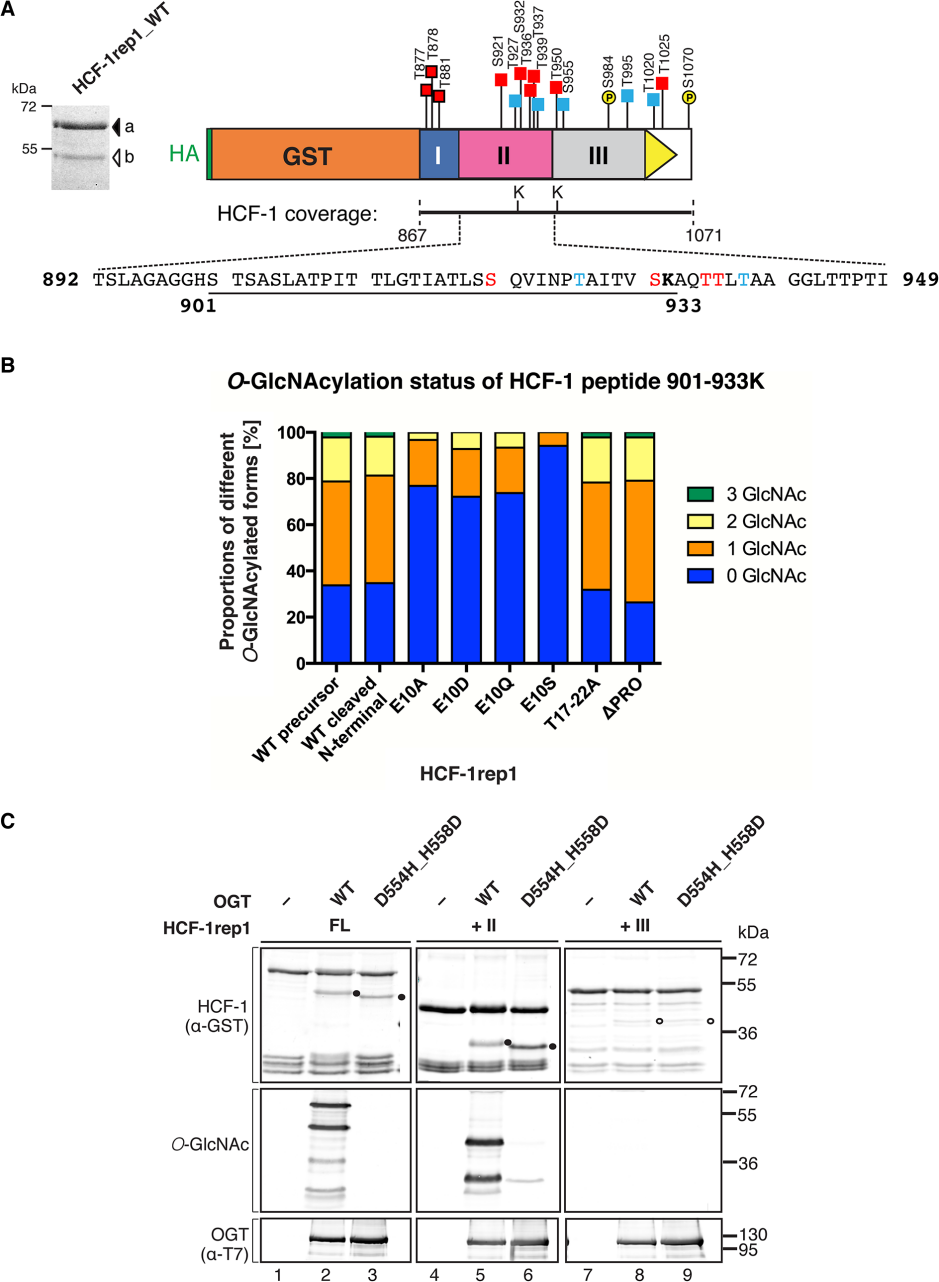
Region II CEE *O*-GlcNAcylation and HCF-1_PRO_-repeat proteolysis are independent OGT activities. (A) (Left) The full-length (FL) HCF-1rep1 precursor (band a) and the N-terminal cleavage product (band b) were purified from HEK 293 lysates via α-HA-epitope immunoprecipitation and visualized by Coomassie staining. The bands were analyzed for *O*-GlcNAcylation and phosphorylation sites by LC-MS/MS. (Right) Schematic representation of identified HCF-1rep1 *O*-GlcNAcylation (squares) and phosphorylation (yellow circles) sites in the uncleaved HCF-1rep1 precursor. The HCF-1 sequences covered by the analysis (residues 867–1071) and the engineered trypsin cleavage sites A933K and M951K are indicated below the diagram. Red and blue squares indicate confident (Mascot score > 23 & probability of localization > 70%) and potential (Mascot score 14–22 or probability of localization 50–70%) *O*-GlcNAcylation sites, respectively. Squares surrounded in black indicate previously identified sites [9]. The HCF-1 Region II CEE amino acid sequence spanning a peptide sequence used in subsequent analyses (underlined: 901–933K) is shown below the diagram.(B) Analysis of a representative Region II CEE peptide (901–933K sequence shown in A) by LC-MS/MS for proportions of different *O*-GlcNAcylated forms. The proportions of 901–933K peptides containing 0, 1, 2 or 3 attached *O*-GlcNAc moieties are given for each sample in percent. HCF-1rep1 constructs were synthesized in HEK 293 cells and peptides were derived from constructs containing wild-type (WT) or mutated (E10A, E10D, E10Q, E10S, T17–22A) HCF-1_PRO_ repeats, or containing a deletion of the HCF-1_PRO_-repeat sequence (∆PRO). The results with WT precursor, E10A, and E10S were confirmed in a second independent experiment.(C) HCF-1rep1 *O*-GlcNAcylation is not fundamental for HCF-1_PRO_-repeat cleavage. *In vitro* cleavage activities of wild-type OGT (WT) and an *O*-GlcNAcylation compromised OGT mutant (D554H_H558D) on selected HCF-1rep1 substrates. Cleavage and *O*-GlcNAcylation activities of constructs containing the full-length HCF-1rep1 sequence (FL), or the Region II CEE (+II) or Region III (+III) sequences were analyzed by immunoblot using the indicated antibodies. We note that the lack of the OGT D554H_H558D *O*-GlcNAcylation activity results in differential mobility of the HCF-1rep1 cleavage products during electrophoresis. Prominent (●) and faint (⭕) cleavage products are indicated.

We purified full-length HCF-1rep1 with the engineered trypsin cleavage sites after transient synthesis in HEK 293 cells and analyzed the uncleaved precursor band (Fig 4A, band a), as well as the N-terminal cleavage product (band b) for *O*-GlcNAcylation. The identified *O*-GlcNAcylation sites of the HCF-1rep1 precursor and the N-terminal cleavage product were nearly identical (S1 Table), suggesting that HCF-1rep1 *O*-GlcNAcylation has minor effects on proteolysis and vice-versa. Fig 4A illustrates schematically the results of the *O*-GlcNAcylation analysis of the HCF-1rep1 uncleaved precursor protein. Because of the new trypsin cleavage sites, full peptide coverage of the HCF-1 sequence was achieved. Previously identified confident *O*-GlcNAcylation sites (red squares) in Region I [9] were confirmed. A cluster of four novel confident (red squares) and two novel potential (blue squares) *O*-GlcNAcylation sites in the Region II CEE, and one novel confident and two novel potential *O*-GlcNAcylation sites in Region III were identified. Thus, in summary, we conclude that the Region II CEE (i) enhances HCF-1_PRO_-repeat cleavage, (ii) associates efficiently with OGT, and (iii) contains a cluster of *O*-GlcNAcylated serines and threonines.

Since HCF-1 is phosphorylated [31, 32, 33] and interference between *O*-GlcNAcylation and phosphorylation in the same protein has been demonstrated in previous studies (reviewed in [4]), we also searched for phosphorylation sites in the LC-MS/MS analysis (Fig 4A, yellow circles). We thus confirmed a previously mapped phosphorylation site in Region III at S984 [a potential glycogen synthase kinase-3 (GSK-3) phosphorylation site; [30]] and identified a novel phosphorylation site at S1070 in the less-well conserved sequence C-terminal of the HCF-1_PRO_ repeat. In an *in vivo* cleavage assay, both an alanine substitution of S984 to inhibit phosphorylation and an aspartate substitution of S984 to constitutively mimic phosphorylation did not alter *O*-GlcNAcylation levels or cleavage efficiencies of the HCF-1rep1 substrates, as detected by immunoblot (S3B Fig). These results indicate that phosphorylation of S984 in Region III interferes neither with HCF-1_PRO_-repeat cleavage nor HCF-1rep1 *O*-GlcNAcylation. Curiously, however, whereas we detected phosphorylation of S984 in the HCF-1rep1 precursor and cleaved products, we only detected S984 *O*-GlcNAcylation in the cleaved product (S1 Table). We have not investigated further a potential relationship between S984 *O*-GlcNAcylation and HCF-1_PRO_-repeat cleavage.

### Region II CEE *O*-GlcNAcylation is not affected by HCF-1_PRO_-repeat proteolysis

It has been suggested that inhibition of HCF-1_PRO_-repeat proteolysis by E10A mutations in the HCF-1_PRO_ repeat decreases general HCF-1 *O*-GlcNAcylation levels [9, 12]. Thus, we investigated whether Region II *O*-GlcNAcylation is affected by E10A (or related mutations) inhibition of proteolysis. For this purpose, we assessed the *in vivo O*-GlcNAcylation levels of a representative Region II CEE peptide by LC-MS/MS (peptide sequence underlined below the diagram in Fig 4A, HCF-1 sequence 901–933). This analysis showed that the HCF-1 901–933 peptide contains 0, 1, 2 or 3 *O*-GlcNAcylation sites, showing that under the conditions of our assay, this HCF-1 sequence is modified sub-stoichiometrically. The HCF-1 901–933 peptides were either derived from wild-type (WT), or from the cleavage inactive E10A, E10D, E10Q, and E10S HCF-1rep1 mutants which, while inhibiting proteolysis, enhance HCF-1_PRO_-repeat–OGT binding (see Fig 1D and 1E). The proportions of HCF-1 901–933 peptides with the different stoichiometries of *O*-GlcNAcylated forms were assessed (Fig 4B). Cleavage inactive mutants containing the E10 mutations (E10A, E10D, E10Q, and E10S) displayed decreased HCF-1 901–933 *O*-GlcNAcylation levels when compared to peptides derived from WT precursor or cleaved N-terminal proteins (Fig 4B).

We also analyzed Region II peptides derived from the T17–22A mutant that inhibits cleavage through a defective HCF-1pro-repeat–OGT TPR domain interaction [22]. To our surprise, HCF-1 901–933 peptides derived from this mutant displayed nearly identical *O*-GlcNAcylation levels in comparison with peptides derived from WT HCF-1rep1 (Fig 4B). This result suggests that OGT binding to the HCF-1_PRO_ repeat is not required for *O-*GlcNAcylation of HCF-1 sequences. Furthermore, the decreased *O*-GlcNAcylation levels observed in the HCF-1rep1 E10 mutants are specific to mutations at the E10 cleavage site and not to the inhibition of cleavage *per se*. To support these results, we deleted the entire HCF-1_PRO_ repeat in the HCF-1rep1 construct to obtain a mutant called HCF-1rep1 ∆PRO, and found nearly identical *O*-GlcNAcylation levels when compared to WT and T17–22A HCF-1rep1 (Fig 4B). Thus, the E10 missense mutants have a dominant repressive effect on Region II *O*-GlcNAcylation, perhaps by trapping and sequestering OGT at the uncleavable mutant E10 HCF-1_PRO_ repeat. We observed the same repressive effect in an LC-MS/MS analysis of peptides derived from Region I (data not shown), indicating that this effect is not confined to Region II.

### The Region II CEE enhances HCF-1_PRO_-repeat cleavage independently of its *O*-GlcNAcylation status

It has been proposed that HCF-1 *O*-GlcNAcylation and site-specific proteolysis influence each other [12]. As HCF-1_PRO_-repeat cleavage did not influence Region II peptide *O*-GlcNAcylation levels, we investigated conversely, whether HCF-1_PRO_-repeat proteolysis could be influenced by Region II CEE *O*-GlcNAcylation. To address this question, we tested HCF-1_PRO_-repeat proteolysis by an OGT mutant that retains its ability to cleave HCF-1rep1, but is compromised for its *O*-GlcNAcylation activity (D554H_H558D OGT; Kapuria and Herr, unpublished results). We compared cleavage and *O*-GlcNAcylation activities of WT and D554H_H558D OGT on HCF-1rep1 FL or +II and +III substrates (Fig 4C). Cleavage efficiencies of substrates cleaved by WT OGT or by D554H_H558D OGT were nearly identical (Fig 4C, upper panel, lanes 2, 3, 5, 6, 8, and 9). Importantly, whereas *O*-GlcNAcylation was detected in the HCF-1rep1 FL and +II construct when cleaved by WT OGT (lanes 2 and 5, middle panel), highly reduced *O*-GlcNAcylation was observed when cleaved by the D554H_H558D mutant (lanes 3 and 6, middle panel), showing that efficient HCF-1_PRO_-repeat cleavage can occur in the absence of prominent HCF-1rep1 *O*-GlcNAcylation. These results suggest that the Region II CEE enhances HCF-1_PRO_-repeat cleavage independently of its *O*-GlcNAcylation status. Indeed, HCF-1 *O*-GlcNAcylation may not in fact directly influence site-specific proteolysis of HCF-1; the cross-talk detected between HCF-1 *O*-GlcNAcylation and proteolysis [12] may have simply resulted from the fact that the same enzyme is responsible for both activities.

## Discussion

We have investigated HCF-1–OGT interactions that promote HCF-1_PRO_-repeat proteolysis and characterized two main aspects: the OGT interaction (i) with the HCF-1_PRO_ repeat 1 and (ii) with a 58 amino acid sequence lying N-terminal of HCF-1_PRO_ repeat 1 called Region II, which represents a cleavage enhancer element referred to here as a CEE.

### The HCF-1_PRO_-repeat E10 cleavage-site residue strains the substrate in the OGT active site

The HCF-1pro-repeat cleavage region binds in the OGT catalytic domain and forms a binding interface with the nucleotide sugar UDP-GlcNAc [22], but extensive interactions via hydrogen bonds with residues in the OGT catalytic domain have not been identified. We have shown that the amino acid side-chain of the glutamate at position 10 of the HCF-1_PRO_ repeat (E10), surprisingly, exhibits a highly unfavorable interaction with the OGT–UDP-GlcNAc complex, and is in fact the only residue in the HCF-1_PRO_-repeat cleavage region to display this behavior. The OGT–HCF-1_PRO_-repeat binding mode is unusual for protease-substrate binding, as such interactions commonly require complementarity between the protease active site and the sequence surrounding the scissile bond [34]. We hypothesize that, here, the energy involved in binding E10 is used to promote HCF-1_PRO_-repeat cleavage. Whereas the wild-type cleavage region causes strains in the OGT–UDP-GlcNAc–HCF-1_PRO_-repeat complex, the threonine region favors HCF-1_PRO_-repeat–OGT association, presumably to ensure proper accommodation of the cleavage region within the OGT catalytic domain. Perhaps the mechanism of HCF-1 cleavage by OGT can involve substrate strains in the OGT active site, because of the large stabilizing effect of the TPR–threonine region interaction. If true, this fact could explain the need for a very large amino acid signal for proteolysis.

Enzymatic mechanisms involving substrate strains in catalysis have been previously described for bacterial carbon-carbon lyases [35]. These enzymes catalyze the hydrolytic cleavage of either one of the amino acids tryptophan and tyrosine, using substrate strains to increase substrate specificity and catalytic efficiency [35]. Interestingly, the substrate strains originating from the E10 residue in the HCF-1_PRO_-repeat cleavage region are dependent on the sugar moiety of UDP-GlcNAc, indicating that HCF-1 proteolysis is coupled to UDP-GlcNAc availability and maybe indirectly to the cellular nutrient status. Future studies will be needed to understand the mechanisms by which substrate strains in the HCF-1_PRO_-repeat cleavage region contribute to HCF-1 proteolysis.

### A CEE OGT-binding site enhances HCF-1_PRO_-repeat cleavage

HCF-1_PRO_-repeat proteolysis is a slow reaction compared to other proteolytic reactions. For instance, complete casein digestion by trypsin occurs in the range of minutes [36]. In contrast, maximum HCF-1_PRO_-repeat cleavage efficiency (approximately 50%) is reached only after eight hours of incubation with OGT. HCF-1_PRO_-repeat cleavage is even less efficient in the absence of sequences N-terminal of the HCF-1_PRO_ repeat 1 (Regions I, II, and III). As the HCF-1_PRO_ repeat itself, by consisting of 26 amino acids, already represents a large protease recognition sequence, we did not expect additional requirements for efficient proteolysis.

The Region II CEE, a sequence rich in serines and threonines, displays prominent sequence-specific HCF-1_PRO_-repeat cleavage-enhancement activity, which correlates to differing extents with efficient *O*-GlcNAcylation and OGT-binding activities. Whereas it is known that, for example, with Hepatitis C virus, protease activities can be stimulated by so-called cofactor domains of the same protease protein [37], with HCF-1_PRO_-repeat cleavage, a protein sequence derived from the substrate surprisingly stimulates protease activity. We note with interest that, within the gene encoding HCF-1, the Region II CEE lies on one exon and the six HCF-1_PRO_ repeats lie on a separate adjacent exon (see S2A Fig)—perhaps during vertebrate evolution the sequences encoding the Region II CEE and HCF-1_PRO_ repeats were juxtaposed in such a manner as to promote appropriate HCF-1-protein maturation.

Currently, there is no evidence for structured protein domains in the HCF-1 Regions I, II, and III defined here (unpublished observations). In fact, these sequences are predicted to be highly unstructured and could form extensive loops, such that the Region II CEE could, via a loop, approach the first HCF-1_PRO_ repeat. The Region II CEE might thus cause enrichment of OGT at the HCF-1_PRO_ repeat 1 and thereby promote cleavage, perhaps because individual HCF-1_PRO_ repeats do not represent effective OGT-binding sequences.

As the Region II CEE did not display sequence specificity for OGT binding in our Region II_scrambled construct, it is possible that OGT preferentially associates with serine/threonine-rich sequences like Region II without high specificity. Our studies indicate that the OGT TPRs are involved in Region II CEE recognition, but we speculate that OGT–CEE-type binding is distinct from the OGT–HCF-1_PRO_-repeat binding mode, because the latter displays extraordinarily high specificity [22]. Structural biology studies addressing the OGT–Region II CEE binding mode could shed light on the mechanisms of OGT target recognition, as well as on HCF-1_PRO_-repeat cleavage enhancement. Additionally, it will be of interest to determine whether other CEEs exist in HCF-1 and what physiological roles they might have in HCF-1 regulation of cell-cycle progression.

### HCF-1 *O*-GlcNAcylation and proteolysis are independent OGT activities

*O*-GlcNAcylation can be influenced by other post-translational protein modifications, such as phosphorylation or ubiquitylation [4, 38–41]. It has been proposed that there is cross-talk between HCF-1 *O*-GlcNAcylation and proteolysis during HCF-1 maturation [12]. In this case, a change in HCF-1_PRO_-repeat cleavage efficiency should cause changes in HCF-1 *O*-GlcNAcylation levels and vice-versa. We thus hypothesized that Region II CEE *O*-GlcNAcylation could be involved in its cleavage-enhancement activity, and initiated proteomic studies of the substrate, i.e. *in vivo* post-translationally modified HCF-1 proteins, accompanied by *in vitro* analyses, using an *O*-GlcNAcylation compromised OGT mutant (D554H_H558D). Contrary to expectations, HCF-1 *O*-GlcNAcylation and HCF-1_PRO_-repeat proteolysis appear to be two independent OGT activities.

Capotosti et al. [9] reported HCF-1_PRO_-repeat-dependent *O*-GlcNAcylation of HCF-1, based on the finding that a series of E10A mutations in the HCF-1_PRO_ repeats decrease overall HCF-1 *O*-GlcNAcylation levels. Here we have shown, using mass spectrometry analyses, that *O*-GlcNAcylation levels of HCF-1 sequences, encompassing the Region II CEE, are not dependent on the presence of the HCF-1_PRO_ repeat. We have further shown that E10 mutations in the HCF-1_PRO_ repeat cause enhancement of OGT–HCF-1_PRO_-repeat association and a reduction of *O*-GlcNAcylation of HCF-1 sequences upstream of the first HCF-1_PRO_ repeat. We thus hypothesize that an E10-mutated HCF-1_PRO_ repeat traps OGT to its HCF-1_PRO_-repeat binding site and consequently reduces OGT association to the other OGT binding-sites of the HCF-1 protein.

If Region II CEE *O*-GlcNAcylation does not impact HCF-1_PRO_-repeat proteolysis, then why is this sequence extensively *O*-GlcNAcylated? *O*-GlcNAcylation can occur co-translationally [42], and it was proposed that the modification could stabilize highly unstructured protein domains, such as the HCF-1 Basic Region and Regions I, II, and III, during and/or after translation. Moreover, *O*-GlcNAcylation has been implicated in the prevention of protein aggregation [8]. Intriguingly, HCF-1 is synthesized as a long precursor protein that might require stabilization of its unstructured regions, particularly before it is able to form stable HCF-1_N_–HCF-1_C_ subunit association [15]. Further studies addressing the role of *O*-GlcNAcylation for HCF-1 protein stability and integrity could elucidate the functional outcome of HCF-1 *O*-GlcNAcylation.

The finding that OGT, well known for its role in protein *O*-GlcNAcylation, could also represent a protease for HCF-1 maturation [9] was unexpected. Here, we have probed the properties of HCF-1 cleavage by OGT and uncovered two novel findings pertaining to the protease activities. In the first case, the target for proteolysis—the E10 glutamate residue—specifically strains the binding of the HCF-1_PRO_ repeat to OGT. In the second case, an auxiliary sequence—the Region II CEE—enhances proteolysis. Given the unusual nature of OGT cleavage of HCF-1, more unexpected findings concerning the mechanism and regulation of OGT-mediated proteolysis are likely.

## Materials and Methods

### Plasmid constructs

The bacterial expression plasmid pGEX-HCF-1rep1 (encoding HCF-1 amino acids 867–1071) containing the wild-type or E10A HCF-1pro repeat 1 was described in [9]. Deletions of Region I (867–891), Region II (892–949), and Region III (850–1009) were obtained by QuickChange site-directed mutagenesis (Agilent Technologies). The scrambled sequence of Region II was generated by random permutation of the amino acids in Region II and the resulting oligonucleotide sequence with additional BamHI restriction sites at the 5’ and 3’ ends custom synthesized. The fragments were PCR amplified and inserted into the pGEX-HCF-1rep1 vector using the BamHI restriction sites. The mutations E10D, E10Q, E10S, T17–22A (Thr 17, Thr 19, Thr 21, and Thr 22; see [9]), and ∆PRO (deletion of HCF-1 residues 1010–1035) in the HCF-1pro repeat were engineered by QuickChange site-directed mutagenesis. The HCF-1rep1 trypsin cleavage sites A933K and M951K for peptide generation in LC-MS/MS analysis were engineered by site-directed mutagenesis.

For transient expression of the HCF-1rep1 deletion constructs in HEK 293 cells, a PCR fragment encoding the GST–HCF-1rep1 sequence was amplified and inserted into a pCGN vector, using XbaI and KpnI restriction sites. The sequence encoding the POUrep2 protein (Oct-1 sequence 280–439, in which the HCF-1_PRO_ repeat 2 was embedded) was amplified from the pET11c vector and inserted as a GST-fusion protein into the pCGN vector, using KpnI and BamHI restriction sites. Constructs containing Regions II or III upstream of the HCF-1_PRO_ repeat were obtained by overlap extension PCR [43].

The bacterial expression plasmid pET24 containing N-terminal T7 and 8-His tags was described in [44] and [22]. Site-directed mutagenesis was used as described above to generate the D554H_H558D OGT mutant.

### Cell culture and plasmid transfections

HEK 293 cells were grown on plates at 37°C in DMEM with 10% FBS. For plasmid transfection, 10^5^ cells were seeded onto a 10 cm plate in 12 ml of DMEM with 10% FBS, and transfected one and a half days after seeding with 4.8 μg of plasmid and 60 μl of Lipofectamine in Opti-MEM medium as described (Invitrogen).

### Bacterial protein expression

The recombinant HCF-1rep1 protein encoding HCF-1 amino acids 867–1071 and its mutants were verified by sequencing. Proteins were synthesized in *E*. *coli* BL21 (DE3) as a fusion with N-terminal GST and C-terminal 6-Hist tag and purified using Nickel affinity chromatography according to the QIAexpressionist protocol (Qiagen) for native protein purification. Briefly, a starter culture of BL21 (DE3) was grown for 6 h at 37°C. 100 ml Luria Bertani (LB) medium containing 100 μg/ml carbenicillin was inoculated with 1 ml of starter culture and grown over night at 22°C. Protein synthesis was induced the next morning with 0.4mM IPTG at 16°C for 6h to reduce the synthesis of C-terminal truncation products of the GST–fusion protein. The bacterial pellet was resuspended in lysis buffer supplemented with Complete protease inhibitor cocktail (Roche) and 0.1mg/ml lysozyme and incubated for 20 min on ice. The lysate was sonicated 12 times for 10 seconds, cleared at maximum speed for 30 min at 4°C, and incubated for 90 min at 4°C with Ni-NTA agarose superflow resin (Qiagen). The resin was washed three times with wash buffer and eluted in 4 ml elution buffer. The proteins were concentrated in Amicon concentration tubes (Milipore) and dialyzed against PBS supplemented with 1 mM DTT overnight at 4°C. The concentrated dialyzed protein was frozen in liquid nitrogen and stored at-70°C.

Recombinant human OGT was synthesized as a His-tag fusion protein in *E*. *coli* BL21 (DE3) cells as described in [22] with minor modifications: Bacterial cultures were grown in 200 ml volumes at 27°C after diluting an overnight culture 1 to 200 in fresh kanamycin supplemented LB media. Cells were grown to an OD_600_ of 1.1 and the temperature was lowered to 16°C for 30min. Protein expression was induced with 0.4mM IPTG at 16°C for 16h. The bacterial pellet was resuspended in 20 ml lysis buffer (50 mM Tris pH 7.5, 150 mM NaCl, 0.1% Triton X-100, 10% glycerol) supplemented with Complete protease inhibitor cocktail (Roche) and 0.1mg/ml lysozyme final concentration and incubated for 30 min on ice. The lysate was then sonicated 12 times for 10 sec to remove viscosity and ensure further lysis. The lysate was cleared by high-speed centrifugation for 45 min at 4°C and incubated for 90 min at 4°C with Ni-NTA agarose superflow resin (Qiagen). The flow-through was removed and the resin washed with 3 column volumes PBS supplemented with 40mM imidazole. The protein was then eluted in PBS supplemented with 250mM imidazole and the elute concentrated in Amicon concentration tubes (Milipore). The elute was dialyzed against PBS supplemented with 1 mM DTT overnight at 4°C. The concentrated dialyzed protein was supplemented with 1 mM DTT and stored at-70°C.

### HCF-1 cleavage and *O*-GlcNAcylation assays

*In vitro* HCF-1_PRO_-repeat cleavage and HCF-1 *O*-GlcNAcylation assays with bacterially synthesized substrates and OGT were performed in 100 mM HEPES (pH 7.9), 5 mM MgCl_2,_ 20 mM KCl, 5 mM DTT, and 10% sucrose at a final volume of 15 μl at 37°C for the indicated time. For a typical assay, bacterially purified GST–HCF-1rep1 (3 μM; concentration measured by comparative analysis with a protein loading control on Coomassie gel) was incubated with bacterially purified OGT (0.6 μM, concentration measured as described above) in the presence or absence of 1 mM UDP-GlcNAc and the reaction stopped by transfer of the tube to-20°C and boiling in SDS-sample buffer. HCF-1 cleavage and *O*-GlcNAcylation were examined by immunoblot.

*In vivo* HCF-1 cleavage assays were performed as follows: HEK 293 cells were transfected in 10 cm dishes with pCGN vectors encoding the HCF-1rep1 or POUrep2 precursor proteins as described above. 48 hours after transfection, cells were washed in PBS and lysed in 0.5% NP40, 10 mM Tris-Cl (pH 8.0), 150 mM NaCl, 5 mM MgCl_2_ (NP40 buffer [45]) supplemented with Roche Complete protease inhibitors and 10 μg/ml Pefabloc SC (AEBSF from Roche Life Science) final concentration for 20 min on ice. The lysate was cleared in a table centrifuge at maximum speed at 4°C for 20 min and incubated with 30 μl mouse α-HA-conjugated agarose beads (Sigma) overnight at 4°C. Beads were washed four times in 0.5% NP40 buffer, and immunoprecipitated material was eluted in 5 bead volumes of HA-peptide (200 μg/ml), in order to avoid elution of the IgG chains from the antibody conjugate, which would interfere with cleavage band analysis by immunoblot. Samples were analyzed by immunoblot.

### *In vitro* HCF-1–OGT binding assay

GST–HCF-1rep1–OGT pull-down assays were performed as in [22] with a few modifications: Prior to the OGT pull-down, 20 μl of a slurry of α-T7 antibody-conjugated beads (polyclonal from goat, Abcam) were incubated with PBS containing 5% (w/v) bovine serum albumin (BSA) for 1 h at 4°C to decrease non-specific binding of GST–HCF-1rep1 to the agarose beads. Subsequently, the beads were washed extensively in PBS. For the OGT pull-down, 2.5 μg GST–HCF-1rep1 and 5 μg of OGT were pre-incubated in 0.5% NP40 buffer supplemented with 5 mM DTT, in the presence or absence of 1 mM UDP-GlcNAc or UDP, in a rotating incubator for 1 h at 20°C. After incubation, 9% of the reaction was removed as an input control for the OGT pull-down. The washed α-T7 agarose beads were added to the reaction, the volume increased to 500 μl using NP40 buffer and the suspension incubated for 1 h at 20°C. The beads were centrifuged and subsequently washed in NP40 buffer at least three times for 5 min each time at room temperature and the OGT–GST–HCF-1rep1 complexes eluted by boiling for 5 min in 20 μl of 2-fold SDS sample buffer. HCF-1rep1–OGT binding was subsequently analyzed by SDS-PAGE followed by immunoblot.

### Immunoblot and antibodies

For immunoblot analysis, nitrocellulose membranes were incubated for 60 min with 5 ml of LI-COR blocking buffer, followed by incubation with the relevant antibodies in 50% LI-COR blocking buffer and 50% PBST (PBS containing 0.1% Tween 20), as described below, at 4°C over night. The membranes were washed four times and incubated with the appropriate secondary antibodies (dilution 1:10,000) in 50% LI-COR blocking buffer and 50% PBST at room temperature for 45 min. The membranes were extensively washed in PBS containing 0.5% Tween20 and scanned with an Odyssey infrared imager (LI-COR).

Antibodies used to detect GST–HCF-1rep1, GST–POUrep2, *O*-GlcNAc and OGT were as follows: rabbit polyclonal α-GST (1–109) (Santa Cruz) used at 1:1000, mouse monoclonal α-HA-epitope (12CA5; [46]) used at 1:1000, mouse monoclonal (RL2) α-*O*-GlcNAc (Abcam) used at 1:3000, mouse monoclonal α-T7 (Novagen) used at 1:5000.

Quantification of bands on immunoblots was performed using the LI-COR Image Quant quantification software and visualized using GraphPad Prism version 6.0e.

### Sample preparation for *O*-GlcNAcylation analysis by liquid chromatography tandem mass spectrometry (LC-MS/MS)

Sample preparation for *O*-GlcNAcylation analysis by LC-MS/MS was performed as in [9] with modifications. HA-tagged GST–HCF-1rep1 plasmid expression vectors with wild-type or with E10A, E10D, E10Q, E10S, T17–22A, and ∆PRO mutated HCF-1_PRO_ repeats were transfected into eight 15 cm dishes of 293 cells for 48 hours. Cells were lysed in 8 ml NP40 buffer supplemented with 1mM PMSF, 10 μM PUGNAc and Roche Complete protease inhibitors. The lysates were denatured by adding a final concentration of 1% SDS and incubation at 65°C for 10 min. The SDS concentration was adjusted to 0.1% by dilution with NP40 buffer, and lysates were incubated with α-HA agarose beads overnight at 4°C. Immunoprecipitated material was eluted with HA-peptide as described above and the resulting elute concentrated using Amicon concentration tubes (Milipore). For proteomic analysis, see below.

### *O*-GlcNAcylation analysis by LC-MS/MS

For the O-GlcNAcylation analysis, purified proteins were resolved by SDS-PAGE and stained with Coomassie Blue. Gel bands were excised from SDS-PAGE gel and in-gel digested with sequencing-grade trypsin (Promega, Madison, WI, USA) as described [47, 48]. Extracted peptides were then cleaved with Glu-C endoproteinase (Sigma-Aldrich, St. Louis, MO, USA). Data-dependent LC-MS/MS analyses were carried out on a hybrid linear trap LTQ-Orbitrap XL (Thermo Scientific) mass spectrometer interfaced to a nanocapillary HPLC equipped with a C18 reversed-phase column (Thermo Scientific), using CID (collision induced dissociation) mode for MS/MS fragmentation. MS/MS spectra were analyzed using Mascot 2.4 software (Matrix Science, London, UK). Mascot was set up to search a custom-built database containing the sequences of the HCF constructions and of contaminants (enzymes, keratins, etc.). Semi-specific cleavage at K, R (not before P), and at E, D were used as the enzyme definition. Mascot was searched with a fragment ion mass tolerance of 0.50 Da, a parent ion tolerance of 10 ppm, and allowing two missed cleavages. Iodoacetamide and propionamide derivatives of cysteine, deamidation of asparagine and glutamine, phosphorylation of serine and threonine, oxidation of methionine, and addition of acetylhexosamine (HexNAc) to serine and threonine were specified in Mascot as variable modifications. O-HexNAc modified and phosphorylated residues were considered confident if the ion score for the identified peptide was superior to 23 with a site localization score (Mascot Delta Score) higher than 70% and potential if the ion score was between 14 and 22 or localization score between 50 and 70%.

### Molecular dynamics simulations

Molecular Dynamics (MD) simulations were performed using stochastic boundary conditions (SBC) [49], after solvating the systems with a 24 Å radius sphere of TIP3P water molecules, centered on the mutated residue. A 4 Å wide restrained buffer region, coupled to a heat bath using the Langevin equation of motion and a 250 ps^-1^ friction constant [50], was used. Protein atoms outside the buffer region were held fixed. The water molecules were kept within the sphere by the use of a solvent boundary potential [51] and a friction constant of 62 ps^-1^ was applied to the water oxygens [50]. After energy minimization, the system was gradually heated and equilibrated at 300 K during 220 ps, while restraints initially applied on the heavy atoms were progressively removed. The MD production run, during which all atoms in the reaction region were unconstrained, was performed at 300 K for 2 ns. All molecular modeling calculations were performed starting from the high-quality crystal structure of the human OGT bound to the peptide from HCF-1pro repeat 2 (1–26) E10Q and UDP-5SGlcNAc (PDB code 4N3B). This experimental structure was preferred to the lower-quality experimental structure with E10 and UDP-GlcNAc (PDB code 4N3C) to perform molecular modeling calculations. Before MD simulations, the 5S sulfur atom was replaced by an oxygen atom, while the Q10 residue of the HCF-1pro repeat 2 was replaced by a E, D or A residue using the UCSF chimera program.

For the calculation of residue contributions to the binding-free energy, the role of each residue on the HCF-1–OGT binding free energy was estimated according to the MM-GBSA approach [23, 24, 52], using the GB-MV2 implicit solvent model [53, 54]. This method allows the decomposition of the total binding free energy into residue contributions. The contribution of each residue of interest was calculated and averaged over 500 frames regularly extracted along a 2 ns SBC MD simulations centered on it.

Molecular graphics were performed with the UCSF Chimera visualization software [55].

## Supporting Information

S1 Fig Related to Fig 1. (A) Alanine scan of the HCF-1_PRO_-repeat residues P7–T14 in an *in vitro* cleavage assay of HCF-1rep1 constructs. Cleavage was detected using α-GST antibodies. Prominent (●) and faint (⭕) cleavage products are indicated. (B) *In vitro* HCF-1rep1–OGT binding assay in the presence of UDP-GlcNAc. Detection of OGT and HCF-1rep1 was performed using the indicated antibodies. Shown are 100% of OGT pull-down (panels a and b) and 11% of the input (panels c and d). *, IgG heavy chain. (C) *In vitro* HCF-1rep1–OGT binding assay in the presence of UDP. (Top) Gel loadings and antibodies used as described in (B). (Bottom) Quantified HCF-1rep1 binding from the OGT-directed pull-down assay (top). Bands were quantified from the immunoblot as ratio of OGT-bound HCF-1rep1 to total HCF-1rep1 in the assay. Obtained values are presented as log2 fold change relative to wild-type HCF-1rep1–OGT binding. In (B) and (C), strong (●) and weak (⭕) OGT binding is indicated.
(TIF)

S2 Fig Related to Fig 2. (A) Sequence conservation among vertebrate and invertebrate species of the human HCF-1 sequences 867–1098. Six different vertebrate species where HCF-1 is cleaved by OGT: Human, Mouse, *Xenopus tropicalis*, *Xenopus leavis*, *Fugu rubripes* and *Danio rerio* were aligned with two invertebrate species where HCF is cleaved by Taspase1: *Apis mellifera* and *Drosophila melanogaster*, using the Jalview bioinformatics tool [26]. Residues are colored in blue according to conservation following the Blosum62 score. Regions I, II, III, IV, and the HCF-1_PRO_ repeats 1 (rep1) and 2 (rep2) are indicated, and the residues were numbered according to the human HCF-1 sequence. The black arrowheads indicate exon boundaries in the human gene *HCFC1* encoding HCF-1. (B) Schematic of the HCF-1rep1 full-length (FL) construct (residues 867–1071) and the deletion constructs used in this study. Constructs ∆I, ∆II, and ∆III lack Regions I, II or III, respectively. ∆I.II.III and ∆I.II.III/E10A contain a deletion of Regions I, II, and III together. Constructs +I, +II, and +III contain only Region I, II or III, respectively, in addition to rep1 and the C-terminal, less-well conserved sequences of 36 amino acids (Region IV). (C) *In vivo* cleavage activities (48 hours) of HCF-1rep1 deletion constructs from three independent experiments (exp.1, exp. 2, exp. 3). 293 cells were transfected with expression vectors encoding HCF-1rep1 FL or the deletion constructs depicted in (A). Synthesized HCF-1rep1 proteins were immunoprecipitated by an N-terminal HA-tag and assayed for cleavage by visualization and quantification of an α-HA tag immunoblot. Cleavage efficiencies are given as ratios of cleaved product over total of uncleaved and cleaved HCF-1rep1 proteins.
(TIF)

S3 Fig Related to Fig 4. Comparison of *in vivo* cleavage and *O*-GlcNAcylation activities (48 hours) between wild-type (WT) HCF-1rep1 and (A) HCF-1rep1 containing engineered trypsin cleavage sites (A933K_M951K) or (B) HCF-1rep1 containing substitutions of the S984 phosphorylation site by alanine (S984A) and by aspartate (S984D), respectively. Cleavage (upper panels) and *O*-GlcNAcylation (lower panels) were detected using the indicated antibodies. The uncleaved precursors (–) and N-terminal cleavage products (●) are indicated.
(TIF)

S1 Table Identification of *O*-GlcNAcylation and phosphorylation sites of the *in vivo* synthesized uncleaved HCF-1rep1 protein and the N-terminal HCF-1rep1 cleavage product. Residues in red and black are confident *O*-GlcNAcylation and phosphorylation sites (Mascot score > 23 & probability of localization > 70%) respectively, and residues in blue are potential *O*-GlcNAcylation sites (Mascot score 14–22 or probability of localization 50–70%). N/A, not applicable; N/D, not detected
(DOCX)
