# Supplementary figures and images for: Distinct OGT-Binding Sites Promote HCF-1 Cleavage
Source: PLoS One. 2015 Aug 25;10(8):e0136636. doi: 10.1371/journal.pone.0136636 (PMC4549301; doi:10.1371/journal.pone.0136636)

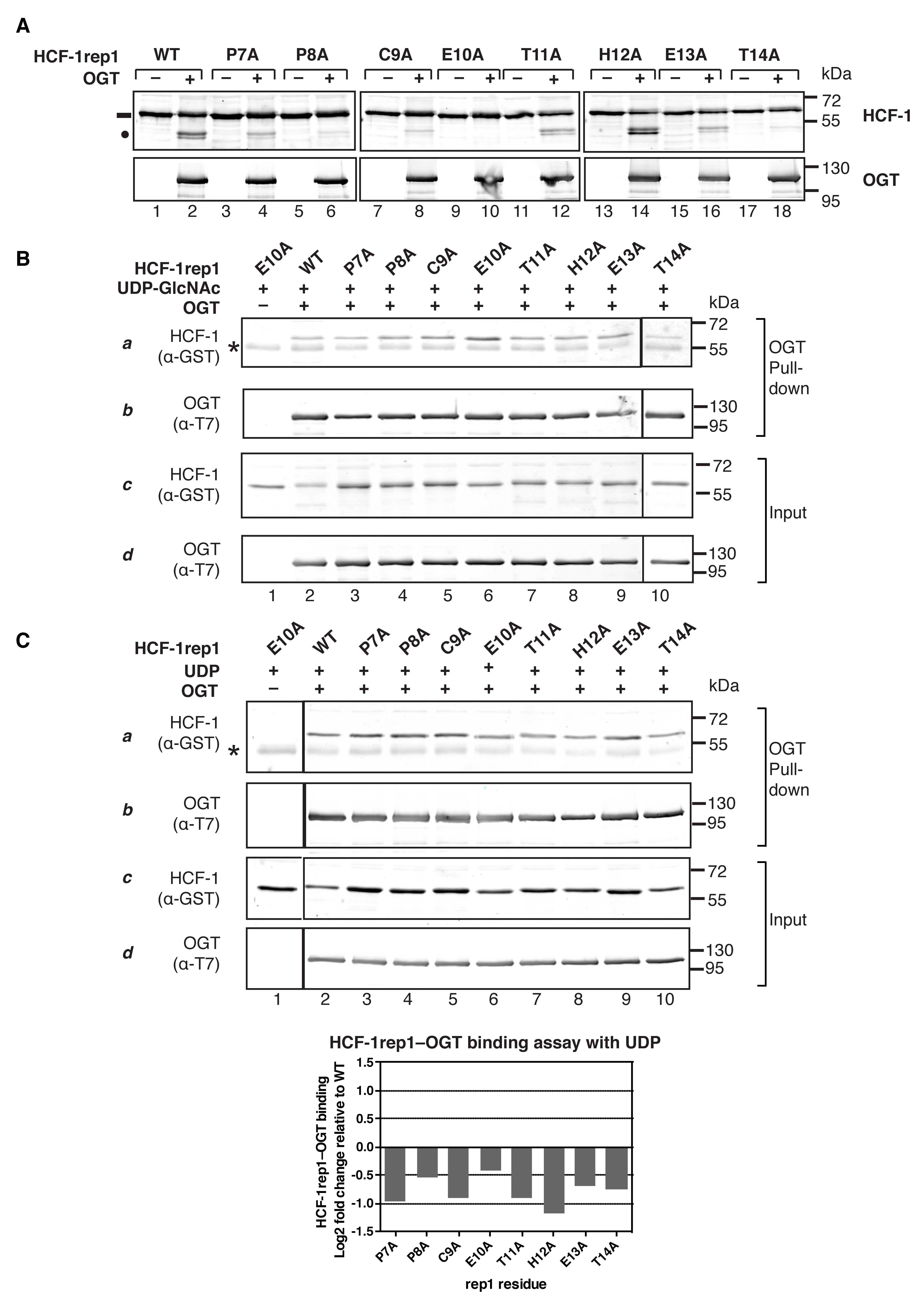

Supplement: S1 Fig — (A) Alanine scan of the HCF-1PRO-repeat residues P7–T14 in an in vitro cleavage assay of HCF-1rep1 constructs. Cleavage was detected using α-GST antibodies. Prominent (●) and faint (⭕) cleavage products are indicated. (B) In vitro HCF-1rep1–OGT binding assay in the presence of UDP-GlcNAc. Detection of OGT and HCF-1rep1 was performed using the indicated antibodies. Shown are 100% of OGT pull-down (panels a and b) and 11% of the input (panels c and d). *, IgG heavy chain. (C) In vitro HCF-1rep1–OGT binding assay in the presence of UDP. (Top) Gel loadings and antibodies used as described in (B). (Bottom) Quantified HCF-1rep1 binding from the OGT-directed pull-down assay (top). Bands were quantified from the immunoblot as ratio of OGT-bound HCF-1rep1 to total HCF-1rep1 in the assay. Obtained values are presented as log2 fold change relative to wild-type HCF-1rep1–OGT binding. In (B) and (C), strong (●) and weak (⭕) OGT binding is indicated. (TIF) [file pone.0136636.s001.tif]

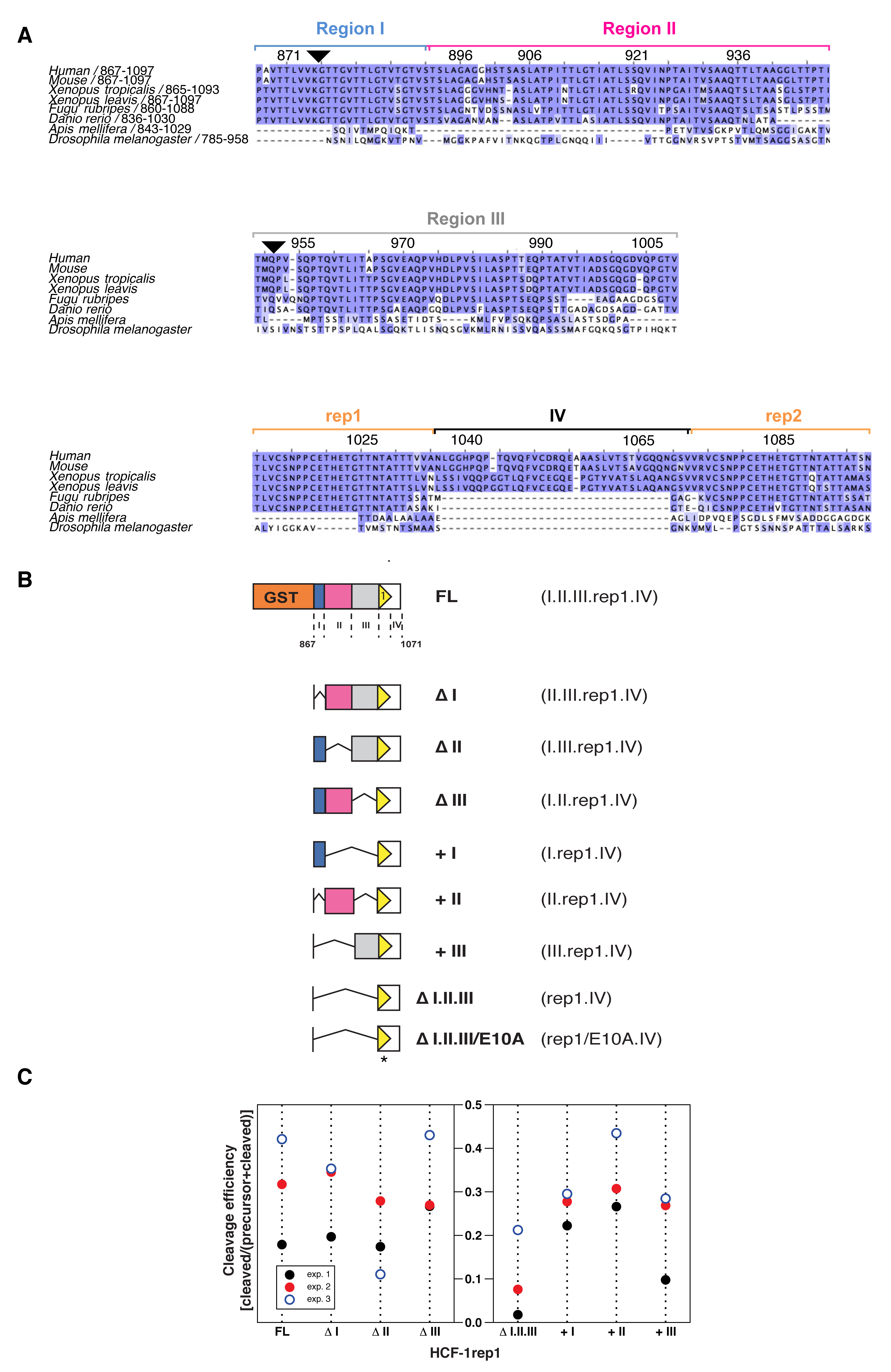

Supplement: S2 Fig — (A) Sequence conservation among vertebrate and invertebrate species of the human HCF-1 sequences 867–1098. Six different vertebrate species where HCF-1 is cleaved by OGT: Human, Mouse, Xenopus tropicalis, Xenopus leavis, Fugu rubripes and Danio rerio were aligned with two invertebrate species where HCF is cleaved by Taspase1: Apis mellifera and Drosophila melanogaster, using the Jalview bioinformatics tool [26]. Residues are colored in blue according to conservation following the Blosum62 score. Regions I, II, III, IV, and the HCF-1PRO repeats 1 (rep1) and 2 (rep2) are indicated, and the residues were numbered according to the human HCF-1 sequence. The black arrowheads indicate exon boundaries in the human gene HCFC1 encoding HCF-1. (B) Schematic of the HCF-1rep1 full-length (FL) construct (residues 867–1071) and the deletion constructs used in this study. Constructs ∆I, ∆II, and ∆III lack Regions I, II or III, respectively. ∆I.II.III and ∆I.II.III/E10A contain a deletion of Regions I, II, and III together. Constructs +I, +II, and +III contain only Region I, II or III, respectively, in addition to rep1 and the C-terminal, less-well conserved sequences of 36 amino acids (Region IV). (C) In vivo cleavage activities (48 hours) of HCF-1rep1 deletion constructs from three independent experiments (exp.1, exp. 2, exp. 3). 293 cells were transfected with expression vectors encoding HCF-1rep1 FL or the deletion constructs depicted in (A). Synthesized HCF-1rep1 proteins were immunoprecipitated by an N-terminal HA-tag and assayed for cleavage by visualization and quantification of an α-HA tag immunoblot. Cleavage efficiencies are given as ratios of cleaved product over total of uncleaved and cleaved HCF-1rep1 proteins. (TIF) [file pone.0136636.s002.tif]

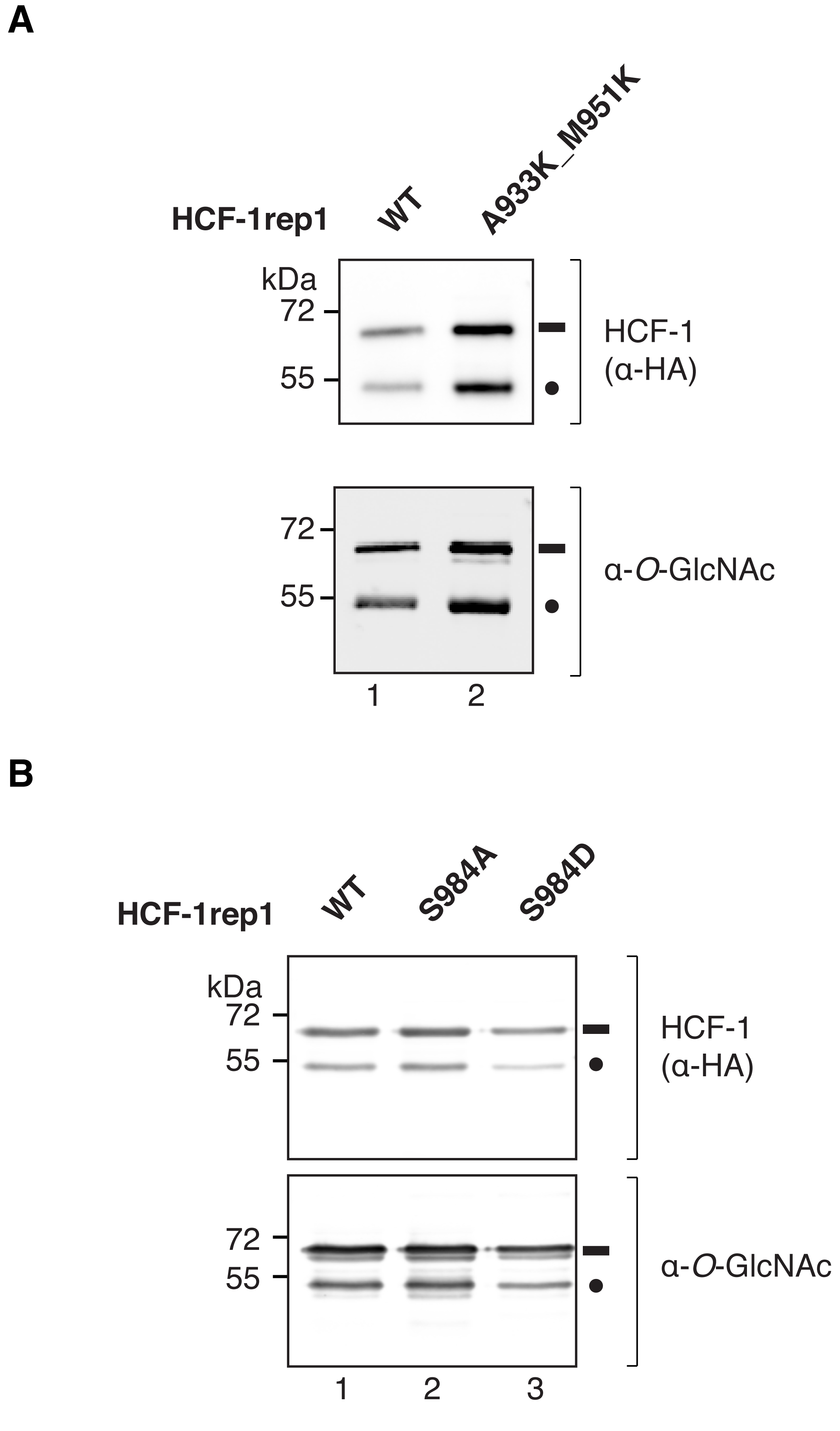

Supplement: S3 Fig — Comparison of in vivo cleavage and O-GlcNAcylation activities (48 hours) between wild-type (WT) HCF-1rep1 and (A) HCF-1rep1 containing engineered trypsin cleavage sites (A933K_M951K) or (B) HCF-1rep1 containing substitutions of the S984 phosphorylation site by alanine (S984A) and by aspartate (S984D), respectively. Cleavage (upper panels) and O-GlcNAcylation (lower panels) were detected using the indicated antibodies. The uncleaved precursors (–) and N-terminal cleavage products (●) are indicated. (TIF) [file pone.0136636.s003.tif]
