# Supplementary material for: Distinct OGT-Binding Sites Promote HCF-1 Cleavage
Source: PLoS One. 2015 Aug 25;10(8):e0136636. doi: 10.1371/journal.pone.0136636 (PMC4549301; doi:10.1371/journal.pone.0136636)
Supplement: S1 Table — Residues in red and black are confident O-GlcNAcylation and phosphorylation sites (Mascot score > 23 & probability of localization > 70%) respectively, and residues in blue are potential O-GlcNAcylation sites (Mascot score 14–22 or probability of localization 50–70%). N/A, not applicable; N/D, not detected (DOCX) [file pone.0136636.s004.docx]

| **Modification** | **HCF-1rep1 uncleaved** | **HCF-1rep1 cleaved** | **Reference** |
| --- | --- | --- | --- |
| *O*-GlcNAcylation | T877 | T877 | Capotosti et al., 2011 |
| *O*-GlcNAcylation | T878 | T878 | Capotosti et al., 2011 |
| *O*-GlcNAcylation | T881 | T881 | Capotosti et al., 2011 |
| *O*-GlcNAcylation | N/D | S901, T902, S903, T905  (ambiguous localization) | novel |
| *O*-GlcNAcylation | N/D | T918 | novel |
| *O*-GlcNAcylation | N/D | S920 | novel |
| *O*-GlcNAcylation | S921 | S921 | novel |
| *O*-GlcNAcylation | T927 | N/D | novel |
| *O*-GlcNAcylation | S932 | S932 | novel |
| *O*-GlcNAcylation | T936 | T936 | novel |
| *O*-GlcNAcylation | T937 | T937 | novel |
| *O*-GlcNAcylation | T939 | T939 | novel |
| *O*-GlcNAcylation | T950 | T950 | novel |
| *O*-GlcNAcylation | S955 | S955 | novel |
| *O*-GlcNAcylation | N/D | S980 | novel |
| Phosphorylation | S984 | S984 | Myers et al., 2013 |
| *O*-GlcNAcylation | N/D | S984 | novel |
| Phosphorylation | T986/T987  (ambiguous localization) | T986/T987  (ambiguous localization) | novel |
| *O*-GlcNAcylation | T995 | T995 | novel |
| *O*-GlcNAcylation | T1020 | N/A | novel |
| *O*-GlcNAcylation | T1025 | N/A | novel |
| Phosphorylation | S1070 | N/A | novel |
